# Load distribution after unilateral condylar fracture with shortening of the ramus: a finite element model study

**DOI:** 10.1186/s13005-023-00370-5

**Published:** 2023-07-08

**Authors:** Loreine M. L. Helmer, Cornelis Klop, Frank Lobbezoo, Jan de Lange, Jan Harm Koolstra, Leander Dubois

**Affiliations:** 1 Department of Oral and Maxillofacial Surgery, Amsterdam Academic Medical Centers and Academic Centre for Dentistry (ACTA), University of Amsterdam and Vrije Universiteit Amsterdam, Amsterdam, The Netherlands; 2 Department of Orofacial Pain and Dysfunction, Academic Centre for Dentistry Amsterdam (ACTA), University of Amsterdam and Vrije Universiteit Amsterdam, Amsterdam, The Netherlands; 3 Department of Functional Anatomy, Academic Centre for Dentistry Amsterdam (ACTA), University of Amsterdam and Vrije Universiteit Amsterdam, Amsterdam, The Netherlands

**Keywords:** Condylar fracture, Finite element model, Load, Resorption, Conservative treatment

## Abstract

**Objectives:**

After a fracture of the condyle, the fractured ramus is often shortened, which causes premature dental contact on the fractured side and a contralateral open bite. The imbalance could change the load in the temporomandibular joints (TMJs). This change could lead to remodelling of the TMJs to compensate for the imbalance in the masticatory system. The load in the non-fractured condyle is expected to increase, and the load in the fractured condyle to decrease.

**Materials and methods:**

These changes cannot be measured in a clinical situation. Therefore a finite element model (FEM) of the masticatory system was used. In the FEM a fractured right condyle with shortening of the ramus was induced, which varied from 2 to 16 mm.

**Results:**

Results show that, with a larger shortening of the ramus, the load in the fractured condyle decreases and the load in the non-fractured condyle increases. In the fractured condyle during closed mouth a major descent in load, hence a cut-off point, was visible between a shortening of 6 mm and 8 mm.

**Conclusions:**

In conclusion, the change of load could be associated with remodelling on both condyles due to shortening of the ramus.

**Clinical relevance:**

The cut-off point implies that shortening over 6 mm could present more difficulty for the body to compensate.

**Supplementary Information:**

The online version contains supplementary material available at 10.1186/s13005-023-00370-5.

## Introduction

A significant impact on the chin can cause a fracture of the condyle. Through the energy transfer, the mandible may fracture directly at the point of the impact or indirectly at the weakest point during force transmission. In the latter case, the kinetic energy is transported through the mandible to the condyle. The condyle can be divided into three parts. The mandibular ramus connects to the condylar base, the condylar neck is in between, and the condylar head is in direct contact with the cartilage of the temporomandibular joint (TMJ). If the impact hits the central part of the symphysis, both condylar heads absorb most of the energy and will fracture [1, 2]. If the direction of the force is more lateral, for example, on the mandibular body or parasymphysis, the weakest point is the condylar neck [1]. After a fracture of the condyle, the traction of the lateral pterygoid muscle induces a shortening of the mandibular ramus on the fractured side, causing premature teeth contact on the fractured side and a contralateral open bite.

Controversies exist about the best treatment modality for a condylar fracture [3, 4]. If a surgeon decides to treat these fractures surgically, the goal of treatment is to recover form and function by restoring the anatomy. In conservative treatment, the focus lies on stabilizing the occlusion and function. For conservative treatment, change of the anatomical shape, in other words, adaptation, of the TMJ is required. However, over 30% of the operated patients also receive treatments that induce adaptation of the TMJ, such as maxillomandibular fixation (MMF) [4]. Thus, open treatment (surgical treatment) of condylar fractures can be seen as mainly anatomy-driven treatment, and closed treatment (conservative treatment) as function-based treatment [5].

Although the anatomy is not restored, the clinical and functional outcome after closed treatment is very similar to that of surgically treated patients [3, 4]. However, there is no fixed protocol for closed treatment of condylar fractures, and the treatment can vary from doing nothing to maxillomandibular fixation [3]. The key to the success of closed treatment remains uncharted territory. From an anatomical perspective, dislocation of the condyle will result in loss of ramus height or a changed angulation of the condyle. Both can cause clinical symptoms, such as malocclusion, visible facial asymmetry, and a deviation to the fractured side during mouth opening. In most cases, after a few weeks of closed treatment, these clinical features have disappeared, but the height of the ramus is still shortened. The anatomical adaptation of the condyle in closed treatment is poorly understood. That is probably the main reason why most surgeons advocate an open approach. Resorption or remodelling is believed to be the pivotal factor in closed treatment.

In 2014, a biomechanical model of the masticatory system was used in a study on condylar fractures. That study showed that after a condylar fracture, movements of the TMJ on the fractured side become limited, and pressure on the articular disc increases, pushing the disc to anterior [6]. This could be related to the change in vertical dimension and, consequently, to a changed load during open and closing movements on the soft and hard tissues within the TMJ. However, the study of Koolstra et al. did not investigate the load in both TMJs during open and closing movement.

Due to the unique way both TMJs are connected by the mandible, the question arises in what way both TMJs influence each other after a condylar fracture. A changed vertical position in the fractured condyle changes the dental occlusion and could also affect the load in the TMJ on the non-fractured side during open and closing movements. Information on this change in load in the TMJs could help to understand the remodelling process after a condylar fracture. Therefore, this paper tries to answer the question of how the load within the TMJ changes on the fractured and the non-fractured side after a condylar fracture. It is hypothesized that after a fracture, the load in the fractured condyle during open and closing movements will decrease, while the load in the non-fractured condyle will increase. There is an expected positive relationship between the amount of ramus shortening on the fractured side and the difference in load between both TMJs during open and closing movements.

## Methods

### The model

The model used for the finite element analysis was a biomechanical model of the human masticatory system constructed in MADYMO (version 7.8, Siemens Industry Software & Services B.V, The Hague, The Netherlands). The model comprises a skull and mandible connected through TMJs. The entire masticatory system was imitated in the model, including bone, cartilage, teeth, muscles, and tendons. The masticatory muscles involved were superficial, deep posterior, and deep anterior masseter, posterior and anterior temporalis, medial pterygoid, inferior and posterior lateral pterygoid, digastric, geniohyoid, and posterior and anterior mylohyoid. The three cartilage layers of the model consisted of 14.500, 12.500, and 12.200 tetrahedral finite elements with edges of about 0.5 mm (HyperMesh 6.0, Altair Engineering GmbH, Böblingen, Germany). The Mooney-Rivlin material model was used to approximate the material properties of cartilage [7]. Data used for the construction of the model was retrieved from human cadavers. For a complete description of the model, see Koolstra and van Eijden [8].

Simulations of open and closing movements were performed as described by Koolstra et al. [6]. The starting position of the model was with closed jaws. One run of the model comprised two entire open-close movements and lasted 0.4 s. At time point zero, the mouth was closed and teeth were in maximal occlusion, which was also the case for time points 185 and 365 ms. At time point 75 and 275 ms, the model reached maximal opening of the mouth, which in case of this model is 30 mm. The movements of the jaws were coordinated by activation of jaw-opening and -closing muscles, imitating a real open- and closing movement.

Each TMJ in the model consisted of three finite element objects: the articulating part of the fossa, the disc, and the articulating part of the condyle. A layer of cartilage material was placed on the fossa and on the condyle, while the disc also consisted of cartilage material. The material properties of the articular surfaces of fossa and condyle were approximated according to the Mooney-Rivlin material model [7], with constants C1 = 4.5 × 10^5^ and C2 = 4.5 × 10^2^ Pa (vide infra) [9]. The material properties of the disc were approximated with use of a non-linear viscoelastic material model, with parameters G_0_ = 0.40 MPa, G_1_ = 0.50 MPa, G_2_ = 0.50 MPa, G_3_ = 0.72 MPa, G_4_ = 2.50 MPa, τ_1_ = 50 s, τ_2_ = 5 s, τ_3_ = 0.2 s, τ_4_ = 0.005 s.

The load output in the condyles, being a result of normal open and closing movements, was subdivided into contact forces and internal forces and was the sum of the elastic, damping, and friction forces. Contact forces were measured on the border tetrahedral elements of the finite element parts of the TMJ. The specific numbers of contact forces per tetrahedral element were combined in a single number per time step for the entire TMJ area. These predictions were represented in graphs showing the mean contact force of the TMJ, i.e., the mean of the contact forces of the superior part of the TMJ, between the fossa and the disc, and the inferior part of the TMJ, between the disc and the condyle. These counts were also summarized in tables. Internal forces were provided by the resultant nodal forces. The internal forces in the condyle were presented visually for each tetrahedral element of the finite element objects using colour maps. These results were analysed and visualized using MADPost (section of MADYMO, version 7.8, Siemens Industry Software & Services B.V, The Hague, The Netherlands).

### Simulations

In this study, the same model was used for all simulations, with different positions of the right condyle. The first set consisted of one run and, with a model without fracture, a symmetrical baseline activity was simulated with a free open-close movement of the mandible. The second set consisted of 8 runs, with a shortening of the right-side ramus, due to the condylar fracture on the right side. The ramus was shortened on an axis between mid-condyle and gonion, i.e., the midpoint of the mandibular angle. The free open-close movement was performed with a shortened ramus of 2, 4, 6, 8, 10, 12, 14, and 16 mm. According to the literature, the maximal deviations used in this FEM to represent the condylar fracture are within the normal range. Condylar fractures can be categorized as minimal (< 2 mm), moderate (2–15 mm), and severe (> 15 mm) [10]. In the results section, only a part of all sets will be depicted. For an overview of all runs, see the Supplementary information.

## Results

**Fig. 1:**
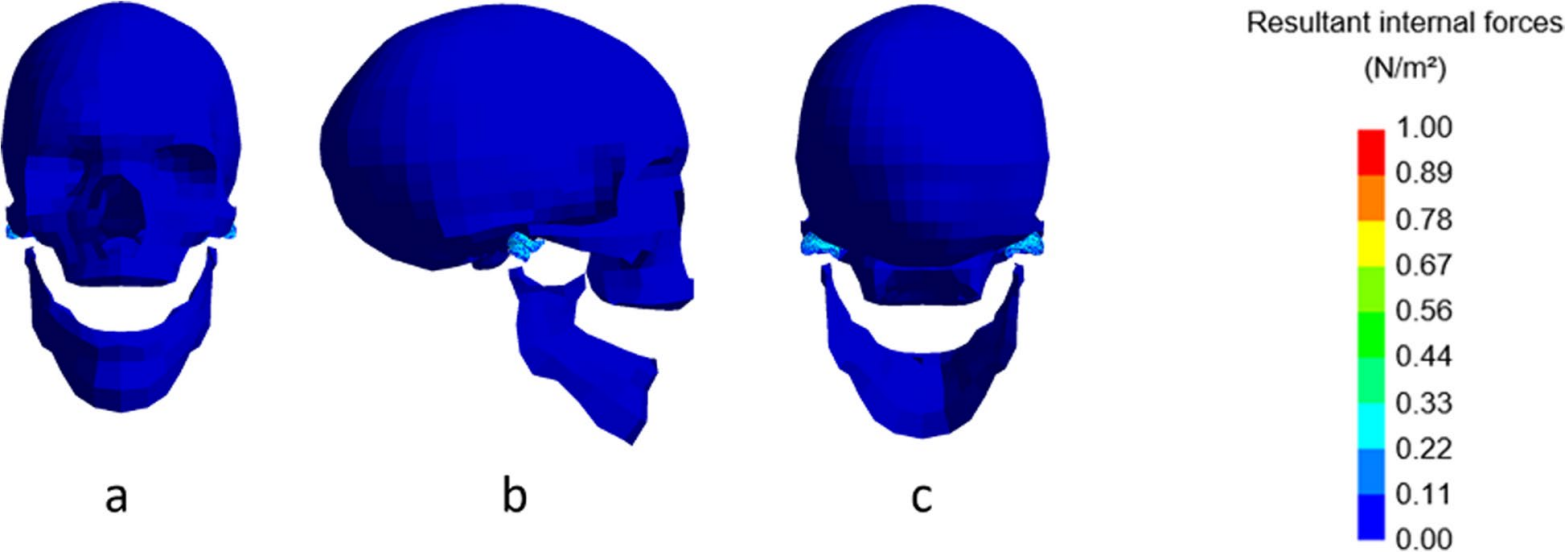
Stress levels in the model at rest. Frontal (**a**), lateral (**b**), and dorsal (**c**) view of the non-fractured model, without fracture, at maximal mouth opening. Inset: color map indicating the stress levels in N/m^2^

In Fig. 1, the main structures of the model without condylar fracture during maximal mouth opening are shown.

The mandible opens symmetrical, and the TMJ areas are coloured from a lighter blue to green, due to the internal forces. To have a better view of the important TMJ-area, the mandible has been displayed as if it were visually disconnected from the skull.

**Fig. 2:**
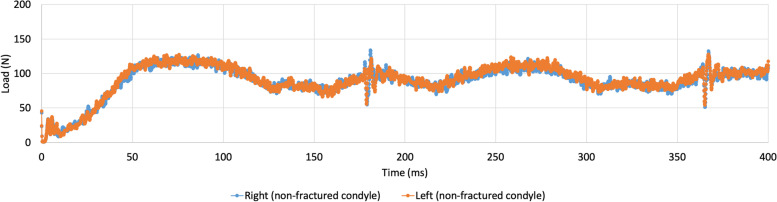
Contact forces in the right (blue) and left (orange) condyle in a model without condylar fracture. Mouth is closed at time points 0, 185, and 365 ms. Maximal mouth opening is reached at time points 75 and 275 ms

This model without any condylar fractures was used for the first set, which consisted of one run of free open-close movements (Fig. 2).

The entire run of two open-close movements took 0.4 s. It is made visible that the load in the condyles during this open-close movement was symmetrical, although the specific values per time step sometimes differed slightly between the right and the left condyle (Fig. 2). As mentioned earlier, at time points zero, 185, and 365 ms, the mouth was closed, and the teeth were in maximal occlusion. This coincides with a lower load on the condyles. At time points 75 and 275 ms, the model reached maximal opening of the mouth, which coincides with a maximum load on the condyles.

## Shortening of the ramus

**Fig. 3:**
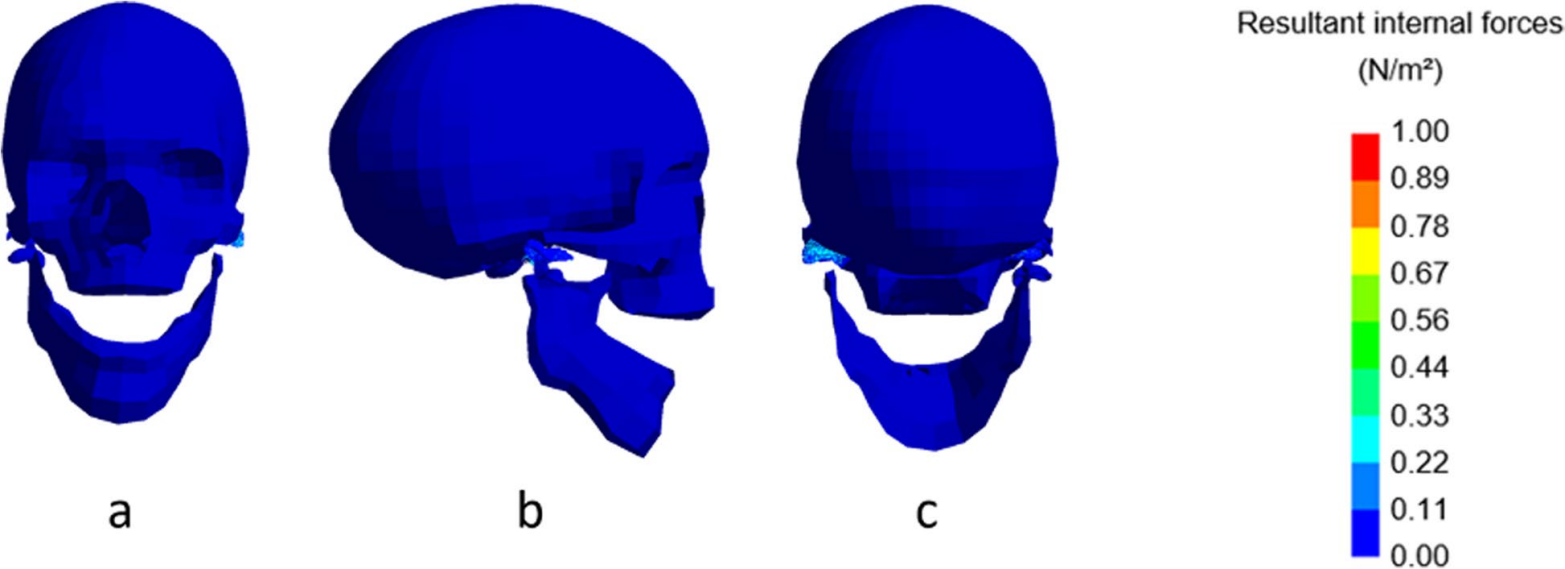
Stress levels in the model at rest. Frontal (**a**), lateral (**b**), and dorsal (**c**) view of the model with fracture and shortening of 16 mm, at maximal mouth opening. Inset: colour map indicating the stress levels in N/m^2^

The second set of condylar fractures introduced in the model was implemented by shortening the ramus on the right side. Shortening of the ramus led to an evident asymmetrical mouth opening with a deviation of the mandible to the fractured side, as seen in Fig. 3.

**Fig. 4:**
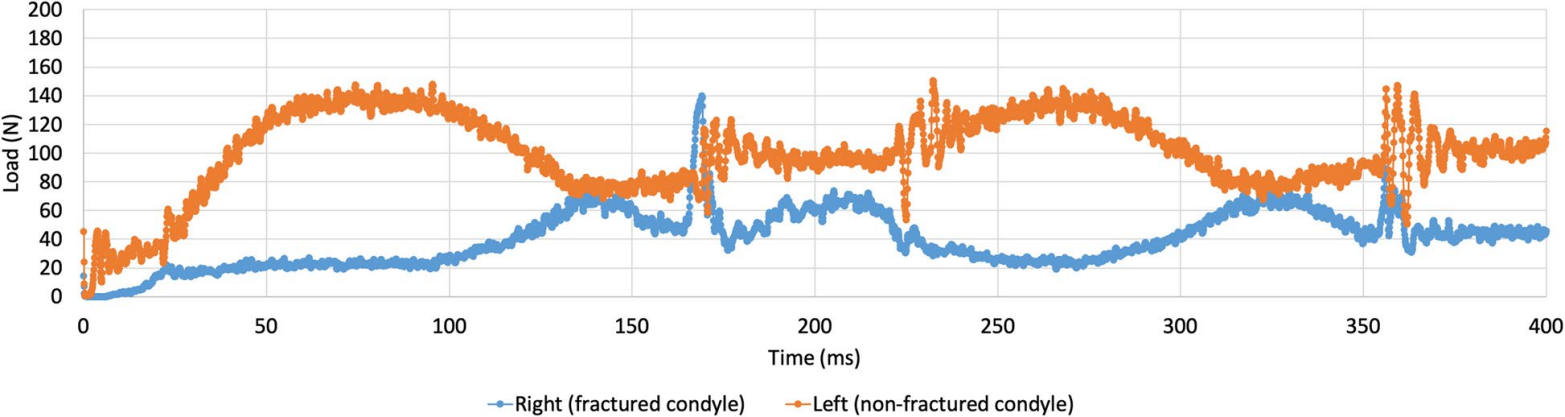
Contact forces in the right, fractured condyle (blue) and left, non-fractured (orange) condyle in a model with condylar fracture and a shortening of 8mm. Mouth is closed at time points 0, 185, and 365 ms. Maximal mouth opening is reached at time points 75 and 275 ms

A fracture of the condyle with shortening of the ramus presented an entirely different output of contact forces compared to the non-fractured model (Fig. 2). This is demonstrated in Fig. 4, where a shortening of 8 mm was implemented.

The contact forces in the left, non-fractured condyle (orange), were much larger than on the fractured right side (blue). This difference was most evident at maximal opening of the mouth (75 and 275 ms).

**Fig. 5:**
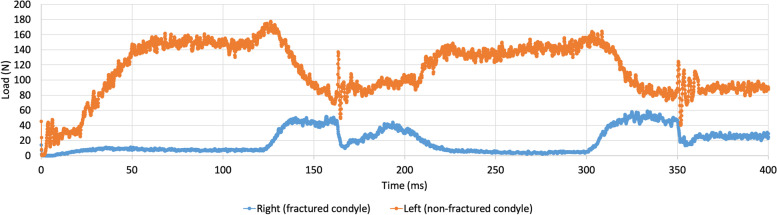
Contact forces in the right, fractured condyle (blue) and left, non-fractured (orange) condyle in a model with condylar fracture and a shortening of 16mm. Mouth is closed at time points 0, 185, and 365 ms. Maximal mouth opening is reached at time points 75 and 275 ms

The same accounted for Fig. 5, where a shortening of 16 mm was implemented.

With a more pronounced difference than Fig. 4, the contact forces in the left, non-fractured, TMJ (orange) were much larger than on the fractured right TMJ (blue). As the figures of the runs in between the non-fractured model, the shortening of 8 mm, and that of 16 mm gradually progressed, these were added to the Supplementary material, Appendix A.

To give some more insight into the proportional differences between the normal run, the shortening of 8 mm, and that of 16 mm, the contact forces were compared in Table 1, an overview of all proportional differences can be found in the Supplementary information Appendix B, Table 2.

**Table 1:** Contact forces in Newton and percentage per TMJ. Shortening of 8 mm and 16 mm compared to the model without fracture

		Jaw closed (time point 180–189.9 ms)	Jaw open (time point 270–279.9 ms)
Without fracture	Right TMJ	99.5 (100%)	107.6 (100%)
	Left TMJ	97.3 (100%)	112.1 (100%)
Shortening 8 mm	Right TMJ/ fractured side	48.6 (48.8%)	24.5 (22.8%)
	Left TMJ/ non-fractured side	99.9 (102.7%)	132.9 (118.6%)
Shortening 16 mm	Right TMJ/ fractured side	30.7 (30.9%)	3.6 (3.3%)
	Left TMJ/ non-fractured side	92.7 (93.2%)	142.5 (127.1%)

**Fig. 6:**
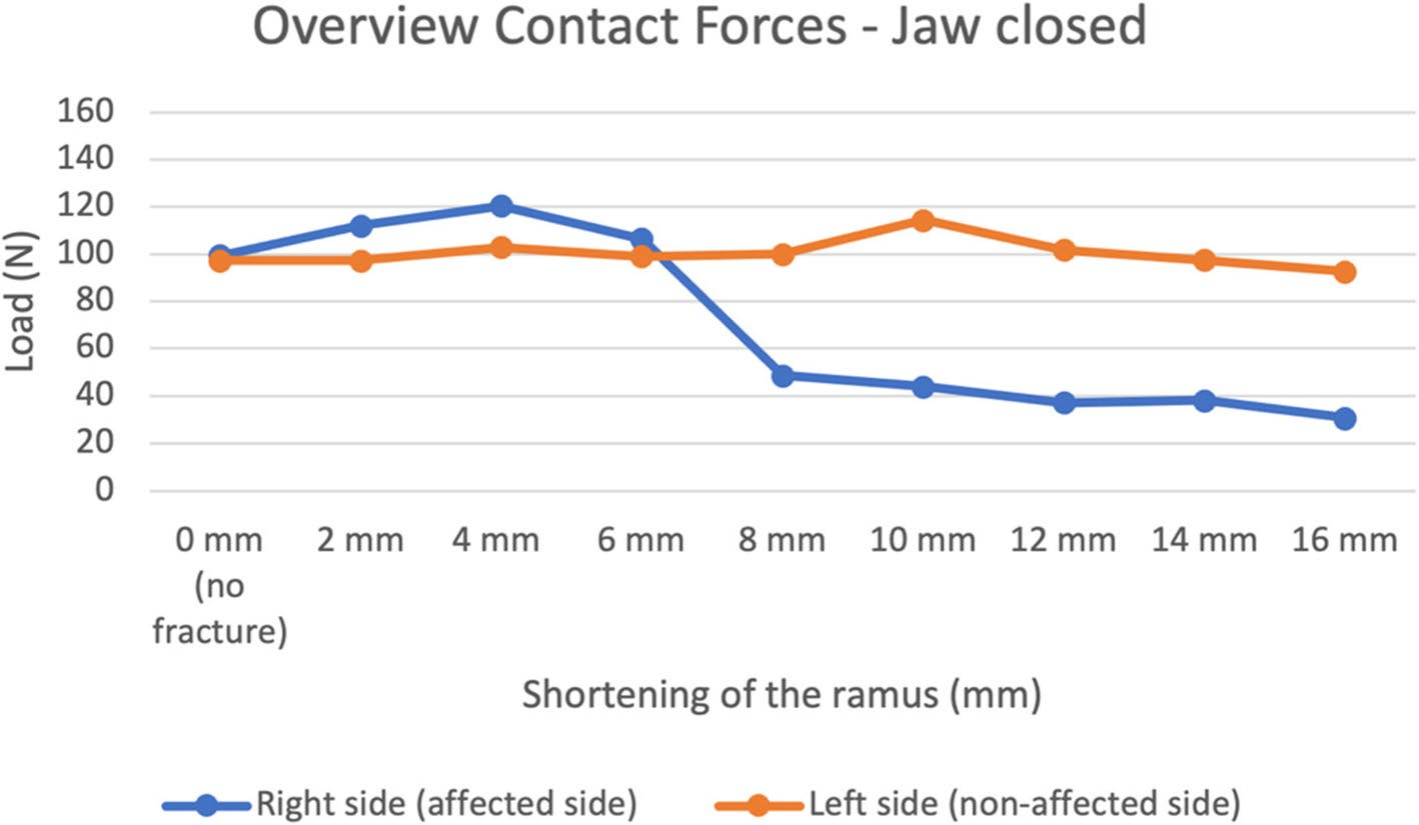
Contact forces in the right, fractured condyle (blue) and left, non-fractured (orange) condyle in all models, during mouth closing

**Fig. 7:**
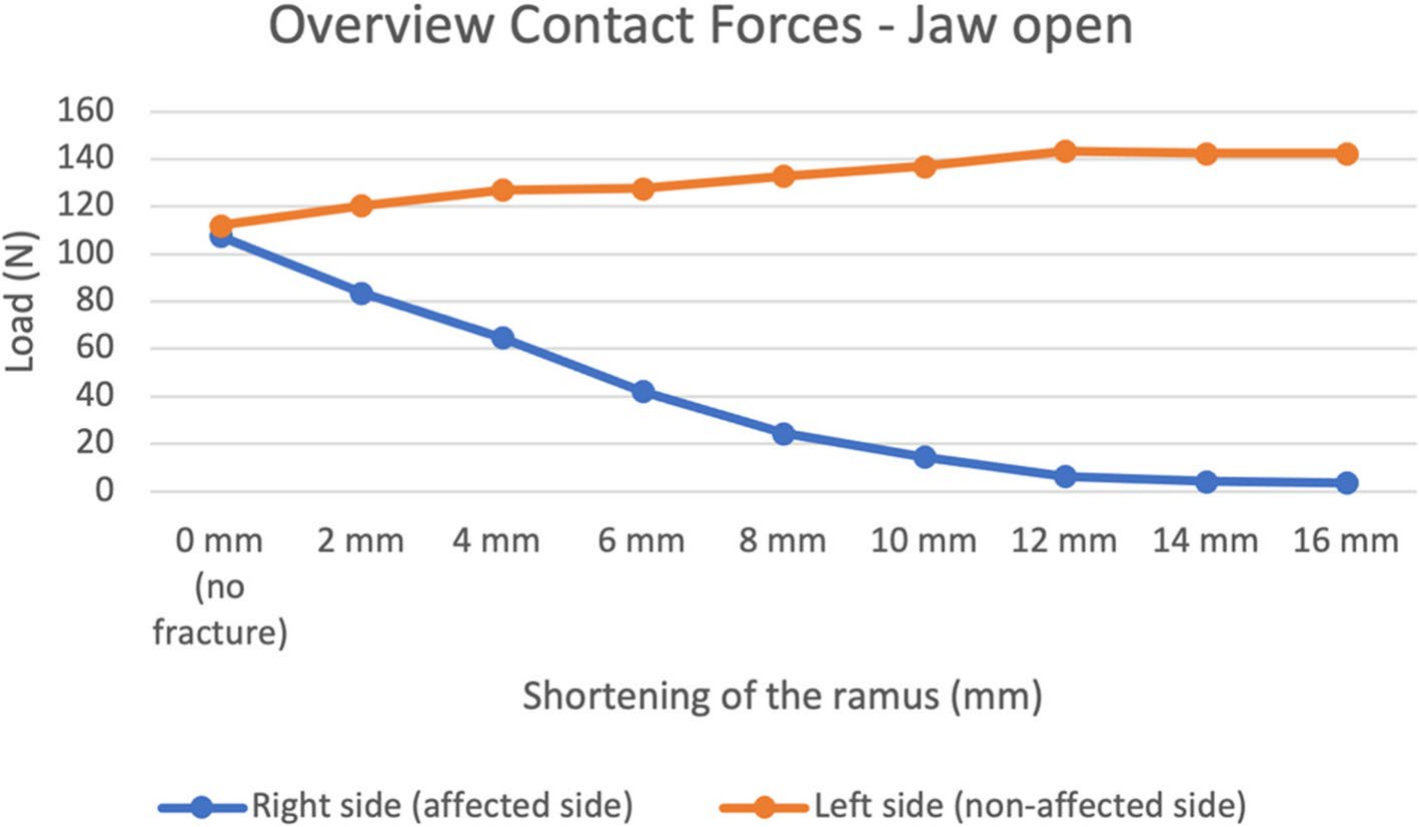
Contact forces in the right, fractured condyle (blue) and left, non-fractured (orange) condyle in all models, during mouth opening

They are also visually represented in Figs. 6 and 7.

Proportional differences between the runs in-between are shown in the Supplementary material. The difference in contact forces between the three runs was most evident during jaw opening. Also, a more extreme shortening of the ramus caused a decrease in contact forces on the fractured side and an increase of contact forces on the non-fractured side.

In Figs. 6 and 7 the mean contact forces from Tables 1 and Table 2 of the Supplementary information are visually represented. A clear pattern is visible in these figures, although fluctuation due to outliers is present, such as the run with 10 mm shortening on the non-fractured side during closed mouth (Fig. 6). Therefore, a trend is harder to describe. Overall, the load on the non-fractured TMJ during closed mouth remains stable, while load on the fractured TMJ decreases. A major change, hence a cut-off point, is visible for the decrease in load on the fractured side, between the shortening of 6 mm and 8 mm. The load in the fractured TMJ between 6 and 8 mm changes from 106.4 N to 48.6 N, which is a decrease of more than 50%. This cut-off point is not visible on the non-fractured side (Fig. 6). During maximal mouth opening, it is clear that load on the fractured side decreases with a more extreme shortening, while the load on the non-fractured increases (Fig. 7).

**Fig. 8:**
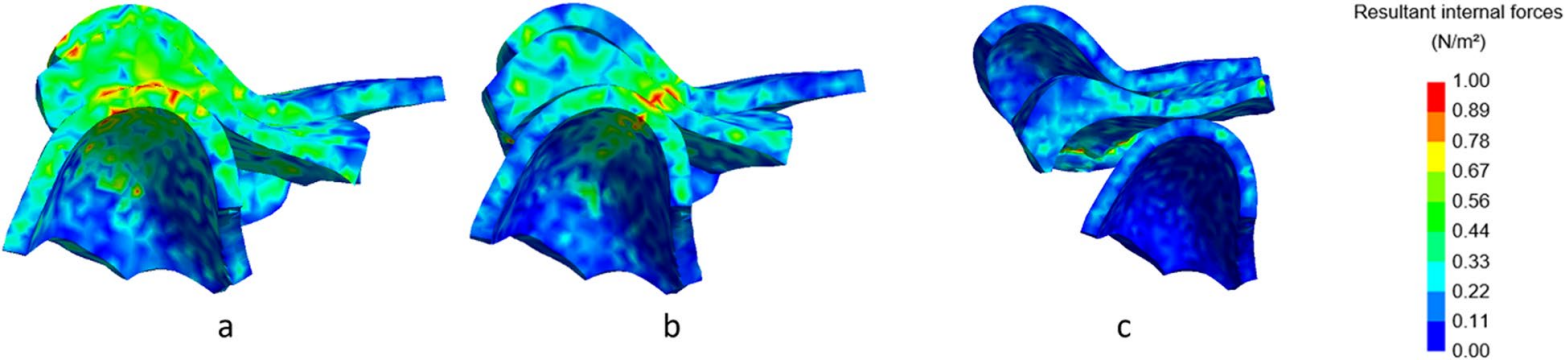
Lateral cross-section of the right TMJ with visualization of the internal forces with closed mouth. No condylar fracture (**a**), condylar fracture right side, shortening of 8 mm (**b**), condylar fracture right side, shortening of 16 mm (**c**). Inset: colour map indicating the stress levels in N/m^2^

**Fig. 9:**
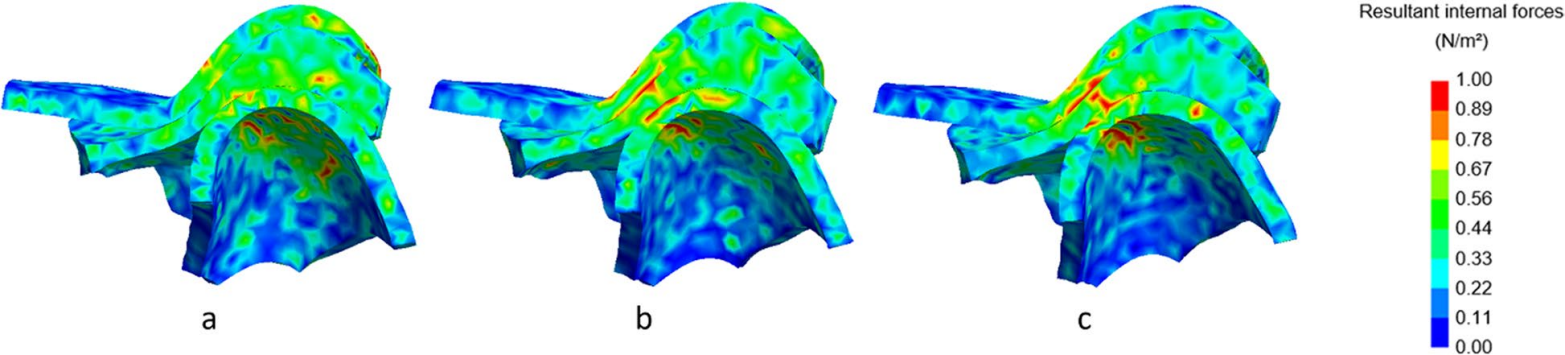
Lateral cross-section of the left TMJ with visualization of the internal forces with a closed mouth. No condylar fracture (**a**), condylar fracture right side, shortening of 8 mm (**b**), condylar fracture right side, shortening of 16 mm (**c**). Inset: colour map indicating the stress levels in N/m^2^

**Fig. 10:**
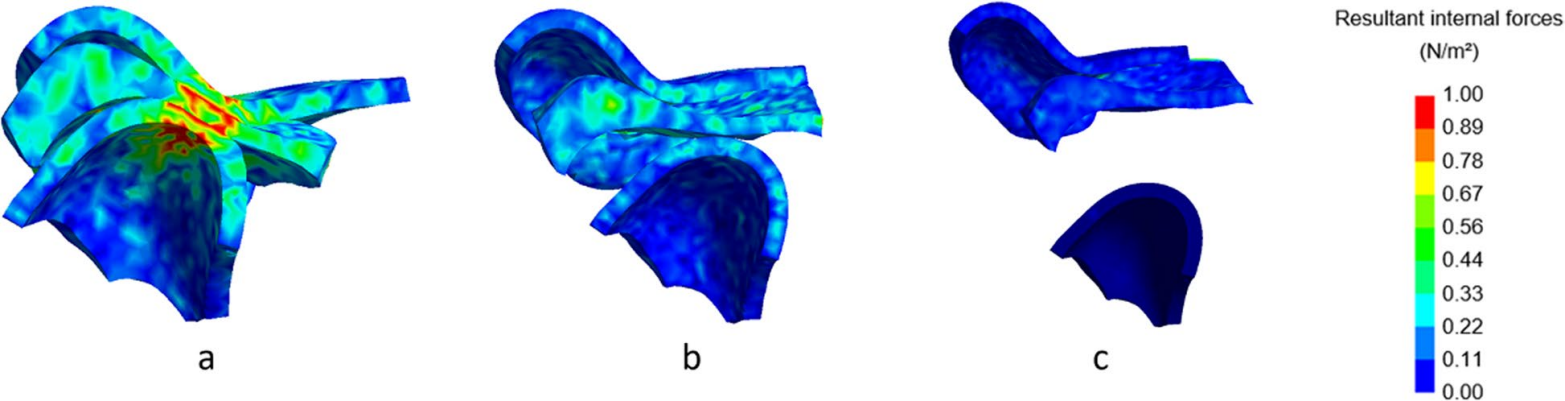
Lateral cross-section of the right TMJ with visualization of the internal forces at maximal mouth opening. No condylar fracture (**a**), condylar fracture right side, shortening of 8 mm (**b**), condylar fracture right side, shortening of 16 mm (**c**). Inset: colour map indicating the stress levels in N/m^2^

**Fig. 11:**
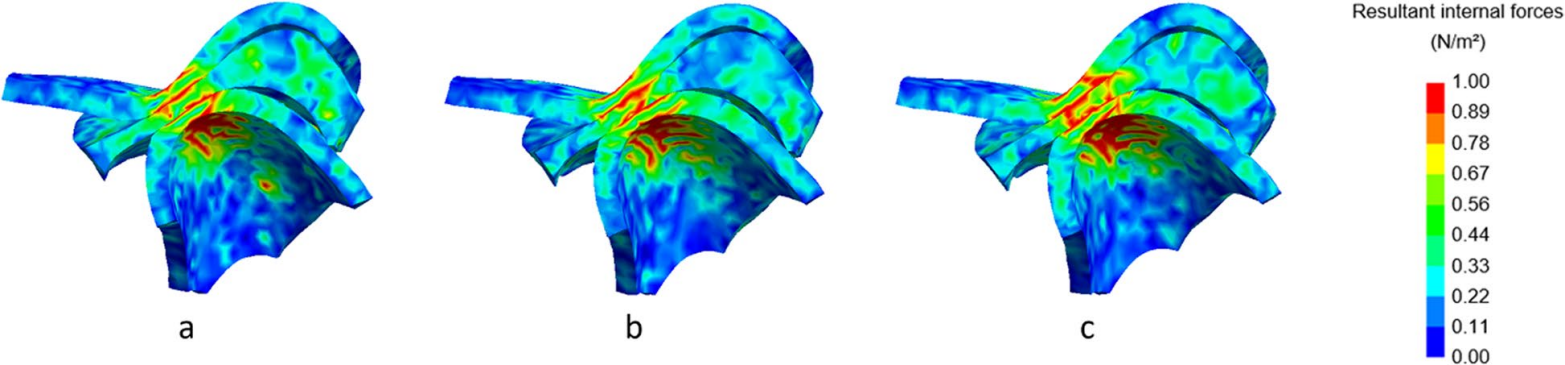
Lateral cross-section of the left TMJ with visualization of the internal forces at maximal mouth opening. No condylar fracture (**a**), condylar fracture right side, shortening of 8 mm (**b**), condylar fracture right side, shortening of 16 mm (**c**). Inset: colour map indicating the stress levels in N/m^2^

Shortening of the ramus also affected the internal forces of the TMJ, as visualized in Figs. 8, 9, 10 and 11. In all Figures, three different finite element parts can be distinguished, the upper part representing the fossa, the middle part representing the disc, and the lower part embodying the condyle.

In Fig. 8 (right condyle) and 9 (left condyle), internal forces of the non-fractured model, without fracture, are compared to the model with shortening of 8 mm and 16 mm at closed mouth (time point 185 ms). In Fig. 10 (right condyle) and 11 (left condyle), internal forces of the non-fractured model, without fracture, are compared to the model with shortening of 8 mm and 16 mm at maximal mouth opening (time point 275 ms).

In line with the results on the contact forces, also the internal forces were smaller on the fractured side (right condyle, Fig. 8). The internal forces of the non-fractured side (left condyle, Fig. 9) increased, while the area where the forces were applied was more concentrated. These differences between fractured and non-fractured side are more evident in Figs. 10 and 11, where the maximal opening of the mouth is shown (time point 275 ms).

Most of the results of the runs with shortening from 2 to 6 mm and 10 to 14 mm were in line with the runs of 8 and 16 mm shortening. Contact forces as well as internal forces changed gradually per run, as can be seen in the Supplementary material. The cut-off point between 6 and 8 mm in the fractured condyle during mouth closing is also visible in the contact forces and the internal forces. For a comparison between 6 and 8 mm of the fractured condyles during mouth closing, see supplementary information (Supplementary information, 22b versus 23b).

## Discussion

This finite element model (FEM) study revealed the unique adaptation mechanism of the body after a fractured condyle. As expected, results showed a change in load not only in the fractured condyle but also in the non-fractured condyle. It was hypothesized that after a fracture, the load in the fractured condyle during open and closing movements would decrease, while the load on the non-fractured condyle would increase. The results confirmed this after shortening of the ramus height. There was an expected inversely proportional relationship between the amount of ramus shortening and the load on the fractured side. The proportional relationship was expected to be positive between the amount of ramus shortening and the load on the non-fractured side during open and closing movements. This was also seen in the comparison between the non-fractured model and the models with shortening of the ramus due to fracture on the right side. The amount of difference in load is not easy to test or quantify. The decrease in load in the fractured condyle after a shortening of 16 mm both in open and closed mouth is evident, but the differences in load on the non-fractured side during closed mouth are harder to define. Possibly these fluctuations can be attributed to inaccuracies in the model, hence for the interpretation of the load, attention was given to numbers that differed more than 10%. Therefore, the sudden drop in load of more than 50% in the fractured condyle during closed mouth between a shortening of 6 mm to 8 mm is considered significant.

This study showed that a fracture that induces shortening of the ramus drastically decreases the load in the fractured condyle during the entire open-close movement. More shortening led to a further decrease in load. In line with the results on the fractured side, the load on the non-fractured condyle increased with shortening of the ramus during the mouth-opening phase. The amount of decrease on the fractured side was not directly compensated by the non-fractured condyle in case of 16 mm shortening. While load increased on the left side by 27%, the decrease on the right side was 3.3%. Possibly, part of this difference is compensated by other structures in the masticatory system, such as muscles.

The sudden drop in load of more than 50% on the fractured condyle during closed mouth is the most significant change in load throughout all results. Whereas all other results show a steady trend with an occasional outlier, the pattern changes significantly between 6 and 8 mm of shortening. Although this does not cause a significant load increase on the other, non-fractured, condyle, it does lead to a sudden large load difference between the condyles and, therefore, a more asymmetric balance. As this sudden increase in difference between condyles is only visible during closed mouth, the role of the dentition should be taken into account. Possibly the stabilization of the dentition has an increasing effect on load of the fractured condyle and a decreasing effect on the load of the non-fractured condyle. However, this cannot entirely explain the sudden drop between 6 and 8 mm. It seems that the two condyles keep working together in distributing the asymmetry in load up to 6 mm. This cut-off point could implicate limitations of recovery of the body if the ramus length asymmetry rises above 6 mm.

In contrast to the load increase in the non-fractured condyle during the mouth-opening phase, the load in this condyle remained stable during the phase of closed mouth. Even during the sudden drop of load in the fractured condyle between the runs with 6 mm and 8 mm shortening, no major change is visible on the non-fractured side. This difference is likely caused by the support of the dentition during the phase of closed mouth. In this FEM periodontal structures have not been taken into account; the teeth are placed in the jaws in an ankylotic form. Literature suggests, however, that tooth, bone, and the periodontal ligament in between react to mastication [11]. Therefore, these structures may have an even greater damping effect on the load in the entire masticatory system, during closed mouth.

The differences in load between the TMJs before and after shortening of the ramus height are substantial. Although the literature shows that the disc plays a major role in the absorption of load in the mandibular TMJ [12, 13], possibly these large load differences can cause clinical sequelae such as pain or impaired function. Remarkably, however, shortening of ramus height due to condylectomy does not show these complications, even with a mean reduction of 8 mm ramus height [14]. Two conclusions can be drawn from this. If a difference in ramus height between the right and left side has a slow onset instead of the rapid change after a fracture, the masticatory system can adapt. Also, the change in ramus height after condylectomy can be compared to the change in ramus height after condylar fracture. In case of condylectomy, the sudden change in ramus height creates a more symmetrical masticatory system, instead of making the system less symmetrical as with unilateral condylar fractures. This implies that not the sudden change in ramus height after condylar fracture is causing complications, but rather the sudden asymmetry in load between the condyles, as is made visible in this study.

The present study investigates load in the condylar TMJ through contact and equivalent stress. The latter is a combined number of different stress-components according to the Von Mises criterion [15]. This represents predominantly shear stress [15]. Since shear does not represent volumetric changes, cartilage handles this type of stress very well [16]. However, results on this type of stress still represent a valuable overview of tensions and deformations that make the cartilaginous structures vulnerable to damage.

Overall, this study showed a fair amount of change in load in the TMJs after shortening of the ramus. The results of this finite element study are in line with the observation that the non-fractured condyle is loaded more heavily during mastication as compared to the fractured condyle [17]. As the results of this FEM study are computed, they cannot be used to draw firm conclusions on clinical outcomes such as pain and remodelling. However, results on changes in load do suggest a possible clinical effect on the surrounding tissue. "Wolff's law" states that mechanical forces guide changes in structure and shape of the bone [18]. Literature has endorsed that disuse of bone will lead to bone loss, whereas mechanical stimulation will promote bone formation [19, 20]. Forces with cyclic impact, like mastication, have a more significant effect on bone formation than a steady high force [21]. If these principles are applied to the results of this study, it would mean that the fractured, shortened side is loaded less, leading to bone loss.

Additionally, the non-fractured side is loaded more, and that would lead to bone formation. However, recent research found that high-intensity mechanical loading induces degradation of bone, instead of formation [22]. Moreover, a higher and longer load leads to more degradation of the bone [23]. The increase of load found in this FEM would lead to degradation of bone on the non-fractured side. Clinical evidence is available that indeed shows a decrease in volume of this non-fractured condylar area [24].

Interestingly, this decrease in volume is not only visible after conservatively treated condylar fractures, but also after surgical treatment [24]. In case of surgery, remodelling could also contribute to the restoration of balance. Another possible explanation for the regain of balance is that soft tissues play a role [24]. A more extreme change in the height of the ramus might not be possible without damage to soft tissues like muscles and tendons, as the damage to the soft tissues is in proportion to the severity of the condylar injury [25]. Also, the cartilage layer has a damping effect on the changes in load, it also shows signs of degradation after high-intensity loading [26].

In conclusion, an increase in load on the non-fractured side could have consequences for the shape of the condyle, in order to regain a balanced distribution of load between the right and left side. As shortening of the ramus due to unilateral condylar fracture causes an increase of the load on the non-fractured side, one would expect more remodelling on the non-fractured side with more shortening. The change of load in the TMJs after shortening is expected to have more effect on the non-fractured side, as this TMJ is loaded up to 27% more after shortening of 16 mm. The change in load through most steps of shortening was gradual. The only cut-off point is the sudden drop in load on the fractured side during closed mouth between 6 and 8 mm shortening. And although the increase in the non-fractured condyle remains proportional, the difference in load between the condyles enlarges significantly. So, this cut-off point may have a significant effect on the remodelling, due to the large difference in load between the condyles. It is also possible that this sudden difference is too large for the body to compensate for, clinical studies could clarify this subject further.

The findings of this study are based on a FEM. Therefore, all results of this study should be interpreted within the boundaries of a FEM. Although the model mimics the masticatory system to a great extent, it should be noted that parts of the model are based on human cadavers. These data may be less representative for mimicking the masticatory system of a healthy adult. Also, the maximal mouth opening of the model was only 30 mm, where in clinical cases a healthy maximal mouth opening for females is around 46 mm and for males around 54 mm [27]. The shortening applied in the fractured condyle is not an imitation of a clinical case, as in patients most shortenings coincide with angulation of the fractured part. By placing a focus on just the shortening, its effect was visible. Before applying these results to clinical cases, it would also be valuable to compare to results with angulation of the fractured condylar part. Apart from the disc, no special attention was given to the soft tissues surrounding the TMJ. These tissues could have a damping effect on contact and/or internal forces of the TMJ. The assumption was that contribution of these soft tissues could be minor, as literature on zygomatic fractures states that soft tissue acts as a temporal buffer only [28]. Future studies could look into the role of soft tissues in condylar fractures. Finally, the implications set in this study by the findings on the cut-off point provide an opportunity for future studies and clinical studies to investigate if an asymmetry between condyles of more than 6 mm leads to more difficulties in clinical situations. And, if so, this could help decisions on treatment of condylar fractures in the future.

## Conclusion

This study provides insight into the changes in load of both TMJs after a unilateral condylar fracture. Shortening of the ramus causes an increase of the load on the non-fractured condyle, and a decrease in load on the fractured side. On the fractured condyle during closed mouth, a significant cut-off point was visible between 6 and 8 mm shortening. This cut-off point could implicate that a larger asymmetry between condyles, than 6 mm, presents more difficulty for the body to adjust. This could have clinical implications for the preferred treatment after condylar fracture.

## Supplementary Information

**Additional file 1: Figure 12.** Contact forces in the right, fractured condyle (blue) and left, non-fractured (orange) condyle in a model with condylar fracture and shortening of 2 mm. Mouth is closed at time points 0, 185 and 365 ms. Maximal mouth opening is reached at time points 75 and 275 ms. **Figure 13. **Contact forces in the right, fractured condyle (blue) and left, non-fractured (orange) condyle in a model with condylar fracture and shortening of 4 mm. Mouth is closed at time points 0, 185 and 365 ms. Maximal mouth opening is reached at time points 75 and 275 ms. **Figure 14. **Contact forces in the right, fractured condyle (blue) and left, non-fractured (orange) condyle in a model with condylar fracture and shortening of 6 mm. Mouth is closed at time points 0, 185 and 365 ms. Maximal mouth opening is reached at time points 75 and 275 ms. **Figure 15. **Contact forces in the right, fractured condyle (blue) and left, non-fractured (orange) condyle in a model with condylar fracture and shortening of 8 mm. Mouth is closed at time points 0, 185 and 365 ms. Maximal mouth opening is reached at time points 75 and 275 ms. **Figure 16. **Contact forces in the right, fractured condyle (blue) and left, non-fractured (orange) condyle in a model with condylar fracture and shortening of 10 mm. Mouth is closed at time points 0, 185 and 365 ms. Maximal mouth opening is reached at time points 75 and 275 ms. **Figure 17. **Contact forces in the right, fractured condyle (blue) and left, non-fractured (orange) condyle in a model with condylar fracture and shortening of 12 mm. Mouth is closed at time points 0, 185 and 365 ms. Maximal mouth opening is reached at time points 75 and 275 ms. **Figure 18. **Contact forces in the right, fractured condyle (blue) and left, non-fractured (orange) condyle in a model with condylar fracture and shortening of 14 mm. Mouth is closed at time points 0, 185 and 365 ms. Maximal mouth opening is reached at time points 75 and 275 ms. **Figure 19. **Contact forces in the right, fractured condyle (blue) and left, non-fractured (orange) condyle in a model with condylar fracture and shortening of 16 mm. Mouth is closed at time points 0, 185 and 365 ms. Maximal mouth opening is reached at time points 75 and 275 ms. **Table 2.** Summery Contact forces. **Figure 20.** Lateral cross-section of the left (a&c) and right (b&d) TMJ withvisualization of the internal forces with closed mouth (a&b) and at maximal mouth opening (c&d). Condylar fracture right side with shortening of 2 mm. Inset: color map indicating the stress levels in N/m^2^. **Figure 21. **Lateral cross-section of the left (a&c) and right (b&d) TMJ with visualization of the internal forces with closed mouth (a&b) and at maximal mouth opening (c&d). Condylar fracture right side with shortening of 4 mm. Inset: color map indicating the stress levels in N/m^2^ . **Figure 22. **Lateral cross-section of the left (a&c) and right (b&d) TMJ with visualization of the internal forces with closed mouth (a&b) and at maximal mouth opening (c&d). Condylar fracture right side with shortening of 6 mm. Inset: color map indicating the stress levels in N/m^2^ . **Figure 23.** Lateral cross-section of the left (a&c) and right (b&d) TMJ with visualization of the internal forces with closed mouth (a&b) and at maximal mouth opening (c&d). Condylar fracture right side with shortening of 8 mm. Inset: color map indicating the stress levels in N/m^2^ . **Figure 24.** Lateral cross-section of the left (a&c) and right (b&d) TMJ with visualization of the internal forces with closed mouth (a&b) and at maximal mouth opening (c&d). Condylar fracture right side with shortening of 10 mm. Inset: color map indicating the stress levels in N/m^2^ . **Figure 25. **Lateral cross-section of the left (a&c) and right (b&d) TMJ with visualization of the internal forces with closed mouth (a&b) and at maximal mouth opening (c&d). Condylar fracture right side with shortening of 12 mm. Inset: color map indicating the stress levels in N/m^2^ . **Figure 26.** Lateral cross-section of the left (a&c) and right (b&d) TMJ with visualization of the internal forces with closed mouth (a&b) and at maximal mouth opening (c&d). Condylar fracture right side with shortening of 14 mm. Inset: color map indicating the stress levels in N/m^2^ . **Figure 27.** Lateral cross-section of the left (a&c) and right (b&d) TMJ with visualization of the internal forces with closed mouth (a&b) and at maximal mouth opening (c&d). Condylar fracture right side with shortening of 16 mm. Inset: color map indicating the stress levels in N/m^2^.

## Data Availability

Availability of data and materials in article supplementary material.
