# Supplementary material for: Load distribution after unilateral condylar fracture with shortening of the ramus: a finite element model study
Source: Head Face Med. 2023 Jul 8;19:27. doi: 10.1186/s13005-023-00370-5 (PMC10329303; doi:10.1186/s13005-023-00370-5)
Supplement: Supplementary file 1 — Additional file 1: Figure 12. Contact forces in the right, fractured condyle (blue) and left, non-fractured (orange) condyle in a model with condylar fracture and shortening of 2 mm. Mouth is closed at time points 0, 185 and 365 ms. Maximal mouth opening is reached at time points 75 and 275 ms. Figure 13. Contact forces in the right, fractured condyle (blue) and left, non-fractured (orange) condyle in a model with condylar fracture and shortening of 4 mm. Mouth is closed at time points 0, 185 and 365 ms. Maximal mouth opening is reached at time points 75 and 275 ms. Figure 14. Contact forces in the right, fractured condyle (blue) and left, non-fractured (orange) condyle in a model with condylar fracture and shortening of 6 mm. Mouth is closed at time points 0, 185 and 365 ms. Maximal mouth opening is reached at time points 75 and 275 ms. Figure 15. Contact forces in the right, fractured condyle (blue) and left, non-fractured (orange) condyle in a model with condylar fracture and shortening of 8 mm. Mouth is closed at time points 0, 185 and 365 ms. Maximal mouth opening is reached at time points 75 and 275 ms. Figure 16. Contact forces in the right, fractured condyle (blue) and left, non-fractured (orange) condyle in a model with condylar fracture and shortening of 10 mm. Mouth is closed at time points 0, 185 and 365 ms. Maximal mouth opening is reached at time points 75 and 275 ms. Figure 17. Contact forces in the right, fractured condyle (blue) and left, non-fractured (orange) condyle in a model with condylar fracture and shortening of 12 mm. Mouth is closed at time points 0, 185 and 365 ms. Maximal mouth opening is reached at time points 75 and 275 ms. Figure 18. Contact forces in the right, fractured condyle (blue) and left, non-fractured (orange) condyle in a model with condylar fracture and shortening of 14 mm. Mouth is closed at time points 0, 185 and 365 ms. Maximal mouth opening is reached at time points 75 and 275 ms. Figure 19. Contact forces in the right, [file 13005_2023_370_MOESM1_ESM.docx]

## Supplementary information, appendix A

### Contact forces Figures 12 - 19
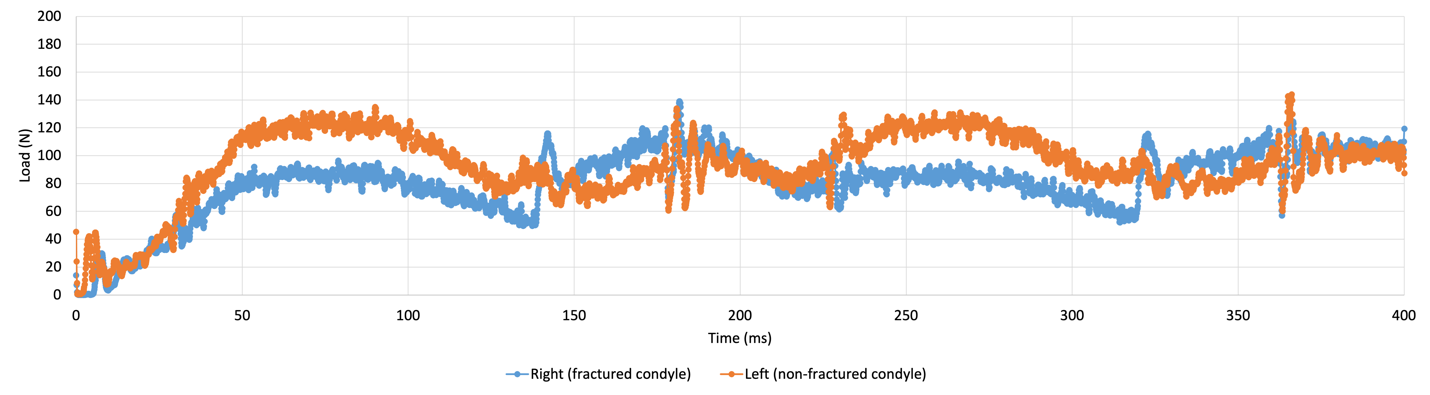


Figure 12. Contact forces in the right, fractured condyle (blue) and left, non-fractured (orange) condyle in a model with condylar fracture and shortening of 2 mm. Mouth is closed at time points 0, 185 and 365 ms. Maximal mouth opening is reached at time points 75 and 275 ms.
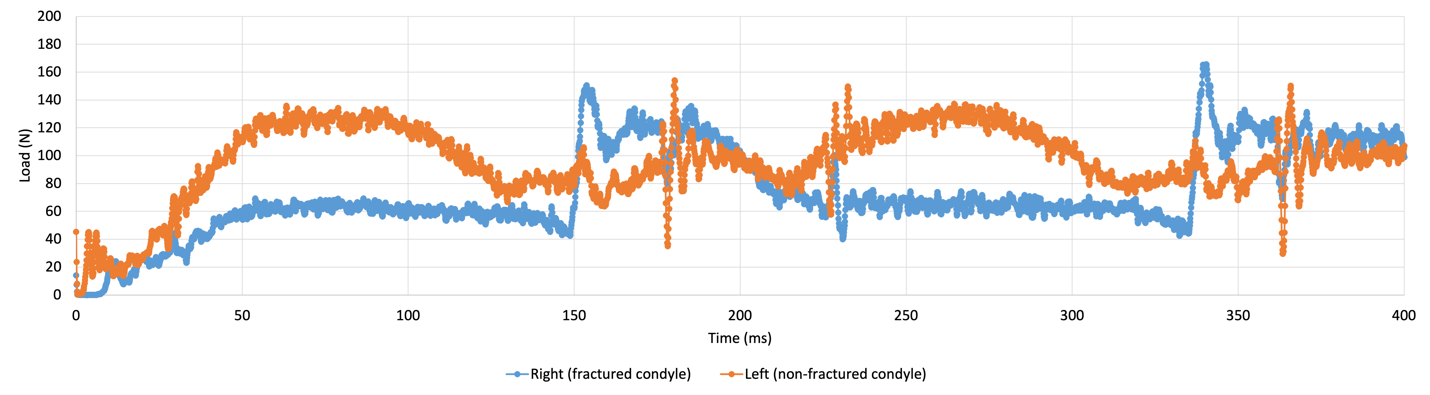


Figure 13. Contact forces in the right, fractured condyle (blue) and left, non-fractured (orange) condyle in a model with condylar fracture and shortening of 4 mm. Mouth is closed at time points 0, 185 and 365 ms. Maximal mouth opening is reached at time points 75 and 275 ms.
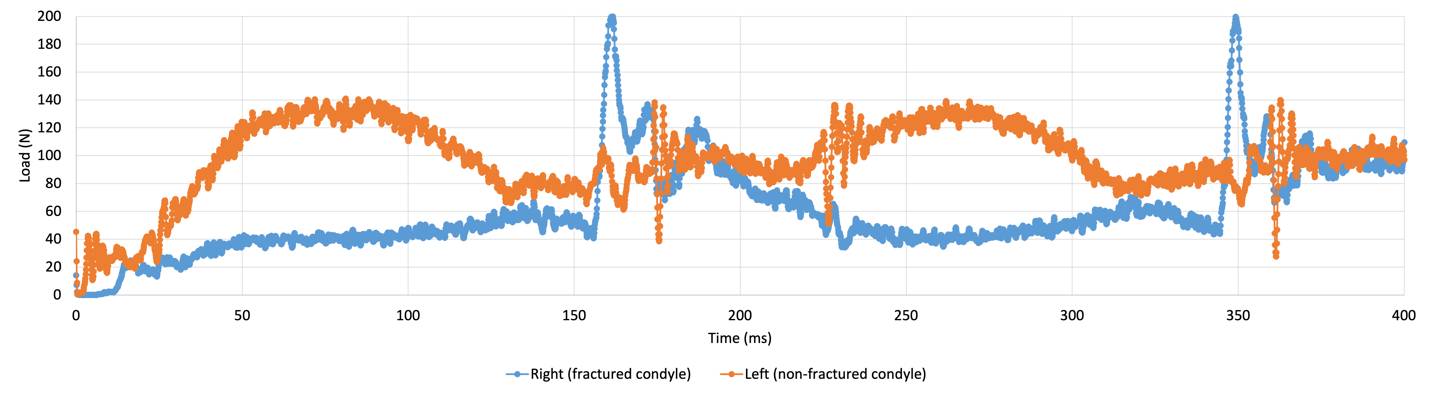


Figure 14. Contact forces in the right, fractured condyle (blue) and left, non-fractured (orange) condyle in a model with condylar fracture and shortening of 6 mm. Mouth is closed at time points 0, 185 and 365 ms. Maximal mouth opening is reached at time points 75 and 275 ms.
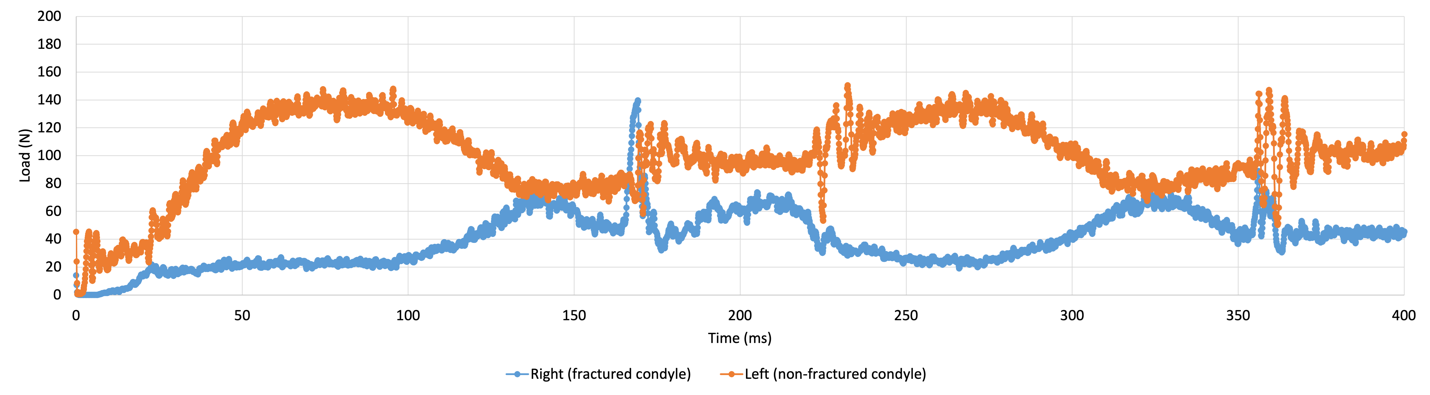


Figure 15. Contact forces in the right, fractured condyle (blue) and left, non-fractured (orange) condyle in a model with condylar fracture and shortening of 8 mm. Mouth is closed at time points 0, 185 and 365 ms. Maximal mouth opening is reached at time points 75 and 275 ms.
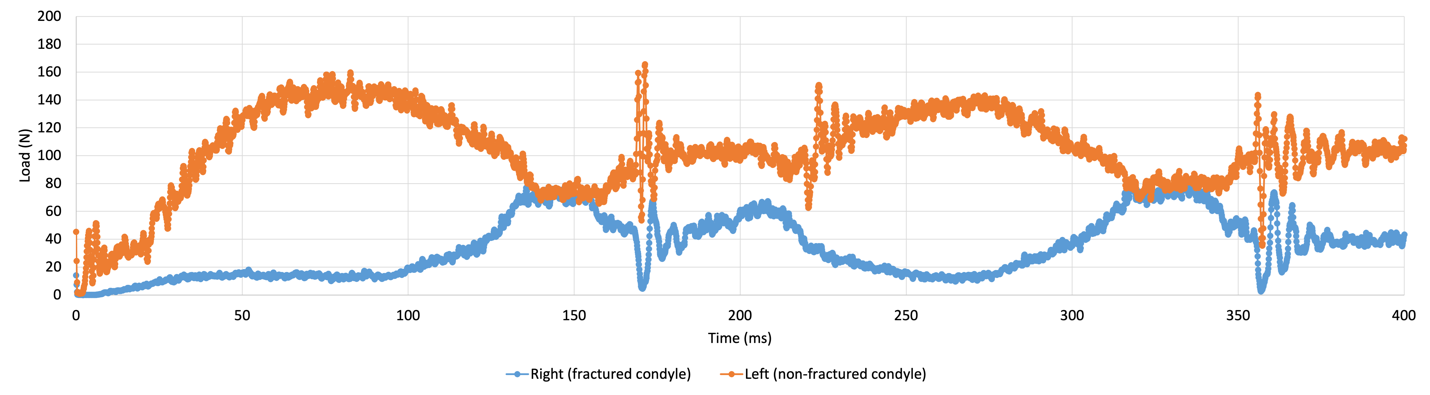


Figure 16. Contact forces in the right, fractured condyle (blue) and left, non-fractured (orange) condyle in a model with condylar fracture and shortening of 10 mm. Mouth is closed at time points 0, 185 and 365 ms. Maximal mouth opening is reached at time points 75 and 275 ms.
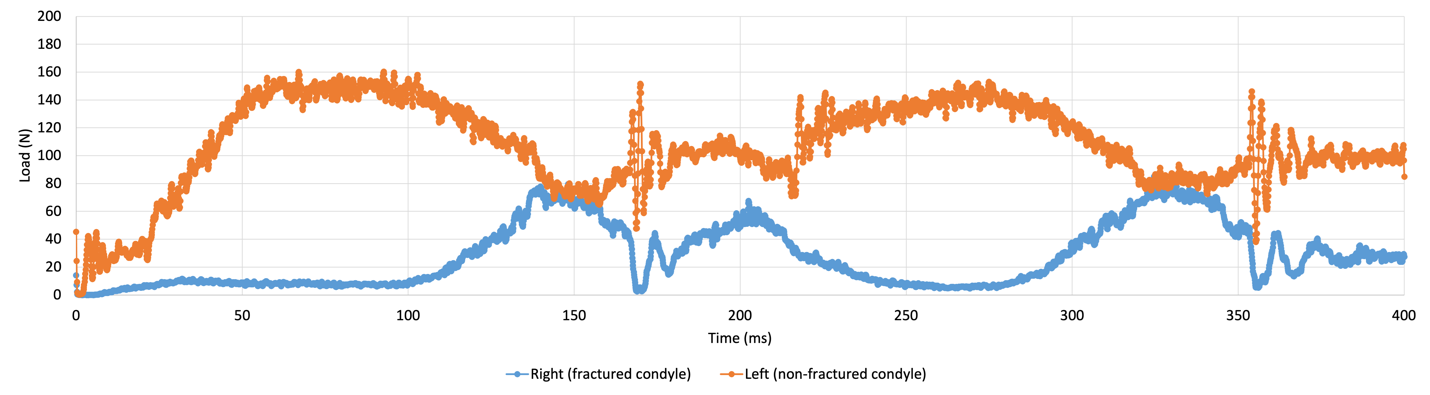


Figure 17. Contact forces in the right, fractured condyle (blue) and left, non-fractured (orange) condyle in a model with condylar fracture and shortening of 12 mm. Mouth is closed at time points 0, 185 and 365 ms. Maximal mouth opening is reached at time points 75 and 275 ms.
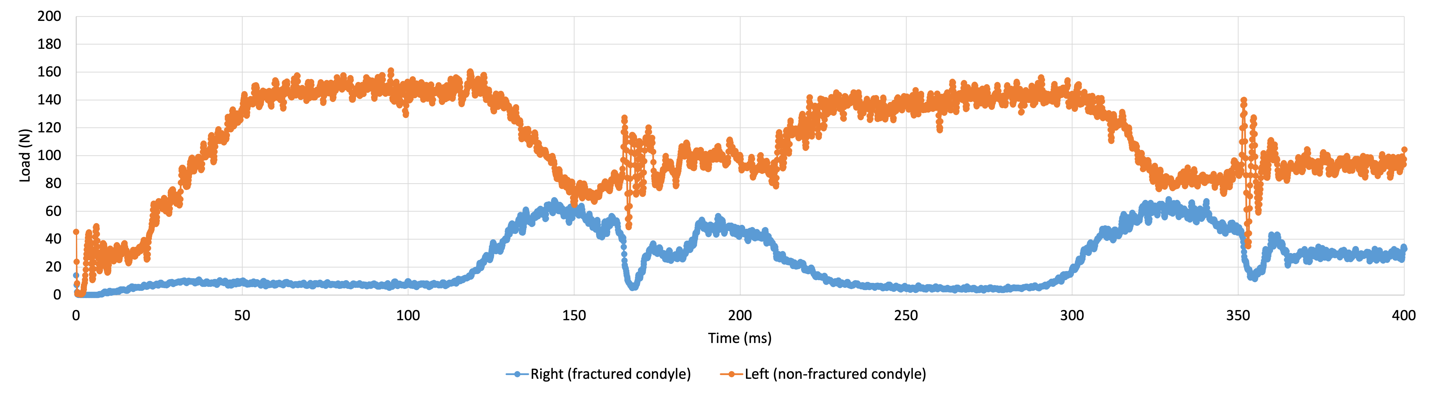


Figure 18. Contact forces in the right, fractured condyle (blue) and left, non-fractured (orange) condyle in a model with condylar fracture and shortening of 14 mm. Mouth is closed at time points 0, 185 and 365 ms. Maximal mouth opening is reached at time points 75 and 275 ms.
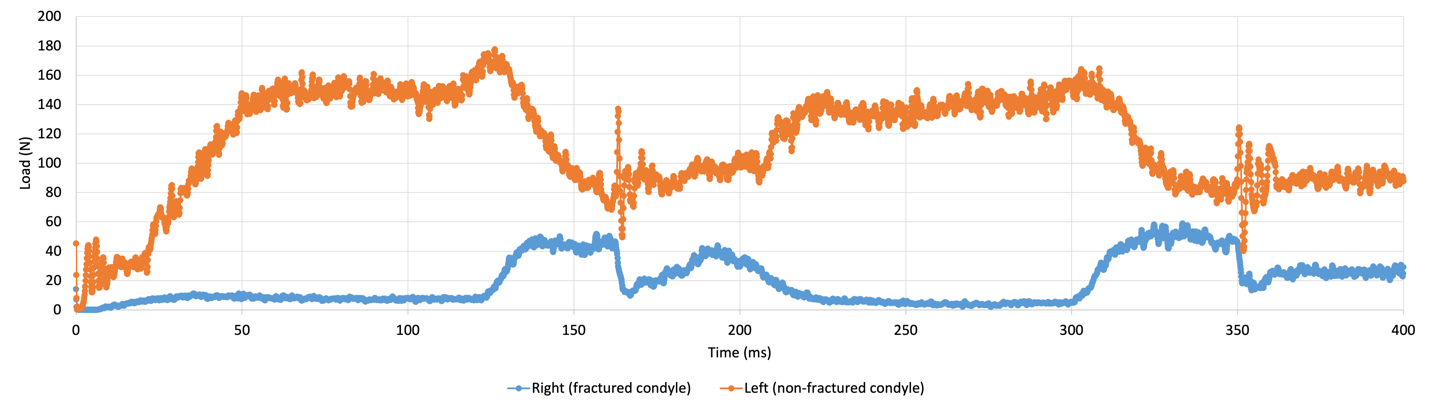

Figure 19. Contact forces in the right, fractured condyle (blue) and left, non-fractured (orange) condyle in a model with condylar fracture and shortening of 16 mm. Mouth is closed at time points 0, 185 and 365 ms. Maximal mouth opening is reached at time points 75 and 275 ms.

## Supplementary information, appendix B

Table 2 Summery Contact forces

|  |  | Jaw closed  (time point 180-189.9 ms) | Jaw open  (time point 270-279.9 ms) |
| --- | --- | --- | --- |
| Without fracture | Right TMJ | 99.5 (100%) | 107.6 (100%) |
|  | Left TMJ | 97.3 (100%) | 112.1 (100%) |
| Shortening 2 mm | Right TMJ/ fractured side | 112.0 (112.6%) | 83.6 (77.7%) |
|  | Left TMJ/ non-fractured side | 97.3 (100%) | 120.5 (107.5%) |
| Shortening 4 mm | Right TMJ/ fractured side | 120.5 (121.1%) | 64.7 (60.1%) |
|  | Left TMJ/ non-fractured side | 103.0 (105.9%) | 127.1 (113.4%) |
| Shortening 6 mm | Right TMJ/ fractured side | 106.4 (106.9%) | 42.2 (39.2%) |
|  | Left TMJ/ non-fractured side | 99.0 (101.7%) | 127.6 (113.8%) |
| Shortening 8 mm | Right TMJ/ fractured side | 48.6 (48.8%) | 24.5 (22.8%) |
|  | Left TMJ/ non-fractured side | 99.9 (102.7%) | 132.9 (118.6%) |
| Shortening 10 mm | Right TMJ/ fractured side | 43.9 (44.1%) | 14.6 (13.6%) |
|  | Left TMJ/ non-fractured side | 114.4 (117.6%) | 137.0 (122.2%) |
| Shortening 12 mm | Right TMJ/ fractured side | 37.2 (37.4%) | 6.4 (5.9%) |
|  | Left TMJ/ non-fractured side | 101.8 (104.6%) | 143.5 (128.0%) |
| Shortening 14 mm | Right TMJ/ fractured side | 38.1 (38.3%) | 4.3 (4.0%) |
|  | Left TMJ/ non-fractured side | 97.4 (100.1%) | 142.4 (127.0%) |
| Shortening 16 mm | Right TMJ/ fractured side | 30.7 (30.9%) | 3.6 (3.3%) |
|  | Left TMJ/ non-fractured side | 92.7 (93.2%) | 142.5 (127.1%) |

## Supplementary information, appendix C

### Internal forces Figures 20 – 27
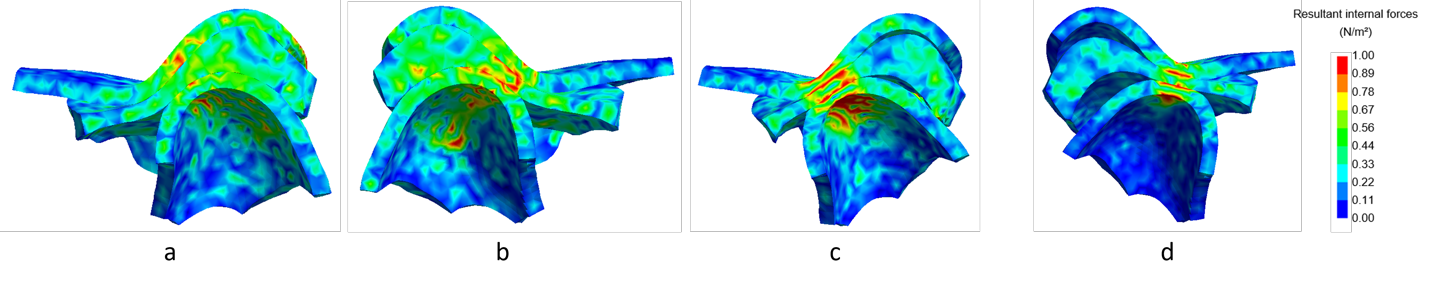


Figure 20. Lateral cross-section of the left (a&c) and right (b&d) TMJ with visualization of the internal forces with closed mouth (a&b) and at maximal mouth opening (c&d). Condylar fracture right side with shortening of 2 mm. Inset: color map indicating the stress levels in N/m^2^.
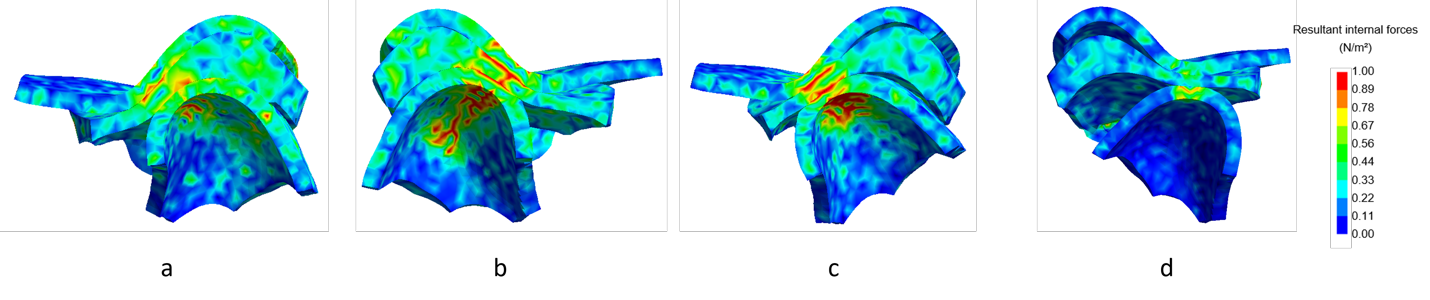


Figure 21. Lateral cross-section of the left (a&c) and right (b&d) TMJ with visualization of the internal forces with closed mouth (a&b) and at maximal mouth opening (c&d). Condylar fracture right side with shortening of 4 mm. Inset: color map indicating the stress levels in N/m^2^.
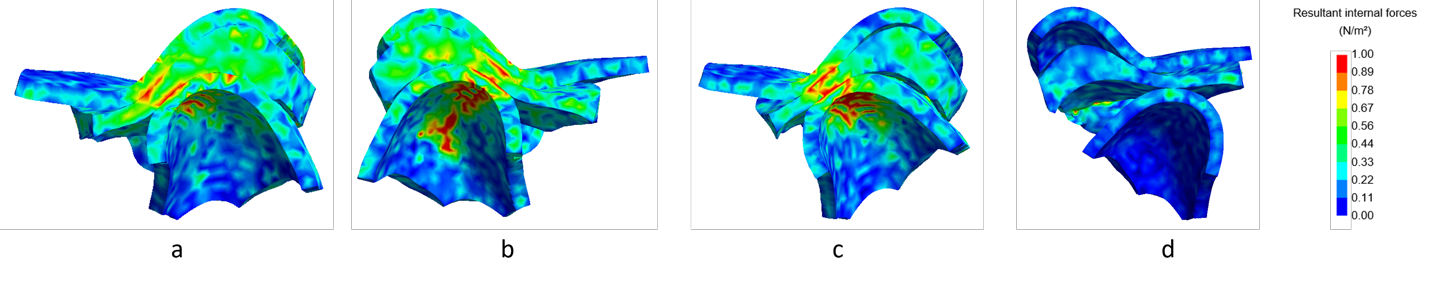


Figure 22. Lateral cross-section of the left (a&c) and right (b&d) TMJ with visualization of the internal forces with closed mouth (a&b) and at maximal mouth opening (c&d). Condylar fracture right side with shortening of 6 mm. Inset: color map indicating the stress levels in N/m^2^.
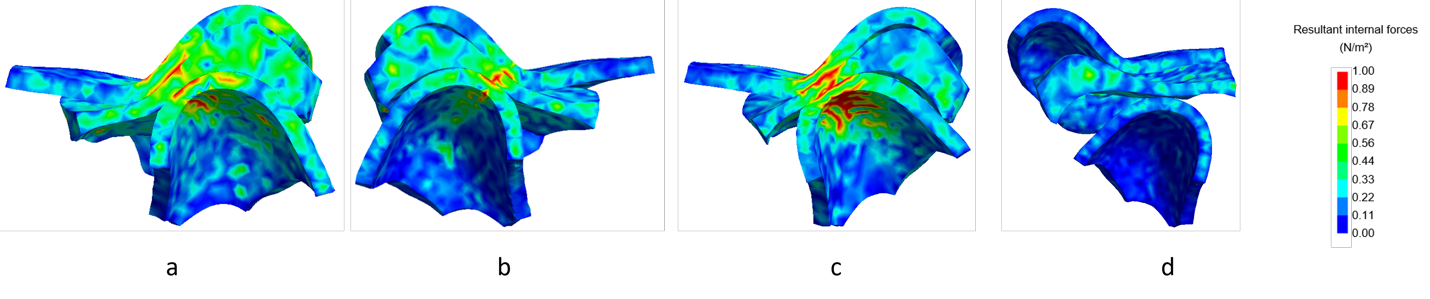


Figure 23. Lateral cross-section of the left (a&c) and right (b&d) TMJ with visualization of the internal forces with closed mouth (a&b) and at maximal mouth opening (c&d). Condylar fracture right side with shortening of 8 mm. Inset: color map indicating the stress levels in N/m^2^.
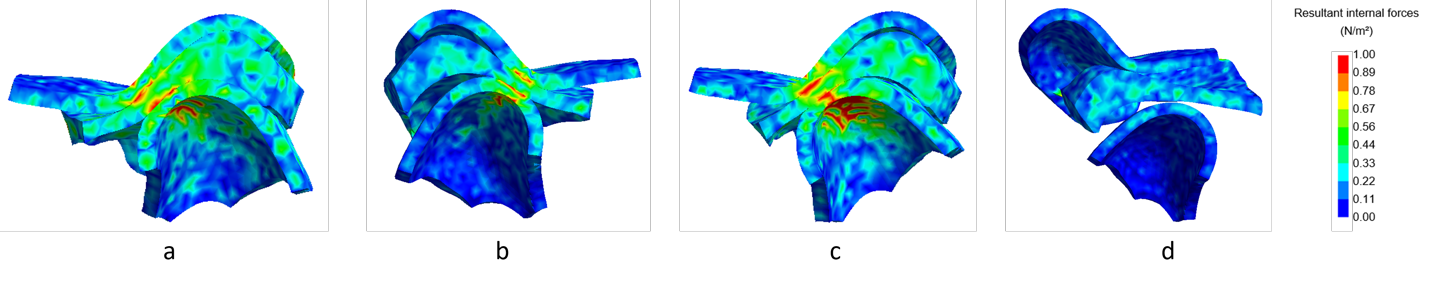


Figure 24. Lateral cross-section of the left (a&c) and right (b&d) TMJ with visualization of the internal forces with closed mouth (a&b) and at maximal mouth opening (c&d). Condylar fracture right side with shortening of 10 mm. Inset: color map indicating the stress levels in N/m^2^.
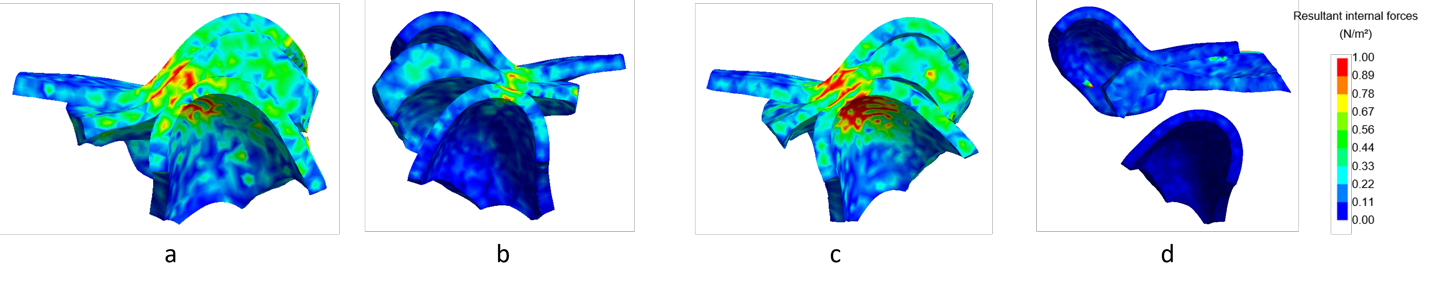


Figure 25. Lateral cross-section of the left (a&c) and right (b&d) TMJ with visualization of the internal forces with closed mouth (a&b) and at maximal mouth opening (c&d). Condylar fracture right side with shortening of 12 mm. Inset: color map indicating the stress levels in N/m^2^.
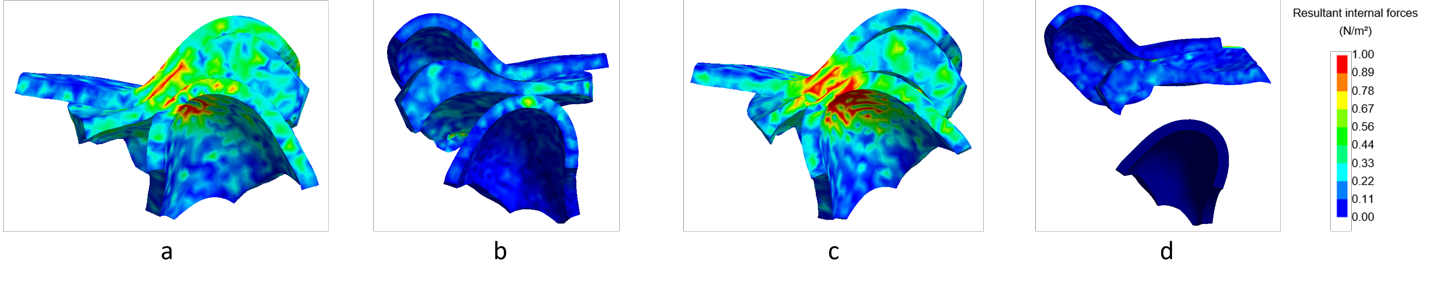


Figure 26. Lateral cross-section of the left (a&c) and right (b&d) TMJ with visualization of the internal forces with closed mouth (a&b) and at maximal mouth opening (c&d). Condylar fracture right side with shortening of 14 mm. Inset: color map indicating the stress levels in N/m^2^.
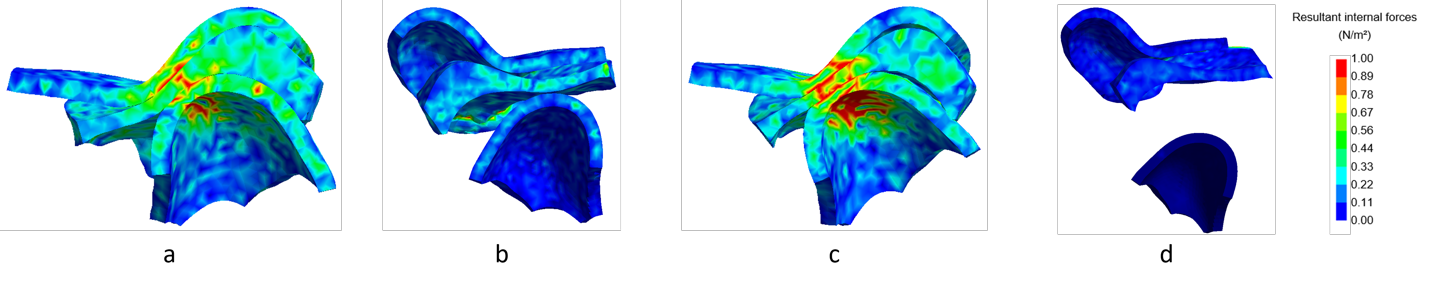


Figure 27. Lateral cross-section of the left (a&c) and right (b&d) TMJ with visualization of the internal forces with closed mouth (a&b) and at maximal mouth opening (c&d). Condylar fracture right side with shortening of 16 mm. Inset: color map indicating the stress levels in N/m^2^.
